# Detection of staphylococcal enterotoxins A and B in cow milk using antigen capture enzyme-linked immunosorbent assay and dot-blot assays

**DOI:** 10.14202/vetworld.2025.686-694

**Published:** 2025-03-23

**Authors:** Hidayatun Nisa Purwanasari, Siti Isrina Oktavia Salasia, Fatkhanuddin Aziz, Rini Widayanti, Madarina Wasissa

**Affiliations:** 1 Department of Clinical Pathology, Faculty of Veterinary Medicine, Universitas Gadjah Mada, Yogyakarta, Indonesia; 2 Department of Bioresources Technology and Veterinary, Vocational College, Universitas Gadjah Mada, Yogyakarta, Indonesia; 3 Department of Biochemistry, Faculty of Veterinary Medicine, Universitas Gadjah Mada, Yogyakarta, Indonesia

**Keywords:** dairy milk, dot-blot, enzyme-linked immunosorbent assay, food safety, staphylococcal enterotoxins A, staphylococcal enterotoxins B, *Staphylococcus aureus*

## Abstract

**Background and Aim:**

*Staphylococcus aureus* is a significant foodborne pathogen responsible for producing enterotoxins, particularly staphylococcal enterotoxins A (SEA) and staphylococcal enterotoxins B (SEB), which are frequently found in milk and dairy products. These toxins in raw milk pose a risk to public health, necessitating accurate and rapid detection methods. This study aimed to develop and evaluate two immunoassays – antigen capture enzyme-linked immunosorbent assay (AC-ELISA) and dot-blot assay – for detecting SEA and SEB in cow milk. The sensitivity and specificity of these assays were compared with the standard polymerase chain reaction (PCR) technique.

**Materials and Methods:**

A total of 30 raw milk samples from Boyolali, Central Java, were subjected to microbiological, genotypic, and immunological analyses. The presence of *S. aureus* was confirmed through culture on Mannitol Salt Agar, biochemical identification, and PCR targeting 23S ribosomal RNA, *nuc*, and *coa* genes. Recombinant SEA and SEB proteins were used to generate polyclonal antibodies for immunoassay development. Dot-blot assays employed nitrocellulose membranes blocked with 1% bovine serum albumin in tris-buffered saline-Tween 20, while AC-ELISA utilized these antibodies for antigen capture. PCR confirmed the presence of the *sea* (127 bp) and *seb* (477 bp) genes. The performance of the immunoassays was statistically evaluated in terms of sensitivity, specificity, and agreement with PCR.

**Results:**

Out of 30 isolates, 27 (90%) were confirmed as *S. aureus*. PCR detected the *sea* and *seb* genes in 23.3% and 30.8% of isolates, respectively. AC-ELISA exhibited sensitivity and specificity of 71.4% and 85% for SEA and 75% and 89.5% for SEB, respectively. The dot-blot assay demonstrated higher sensitivity (85% for SEA and 87.5% for SEB) but comparable specificity (85.7% for SEA and 84.2% for SEB). Kappa values indicated substantial agreement between the immunoassays and PCR results.

**Conclusion:**

Both AC-ELISA and dot-blot assays demonstrated considerable potential for detecting SEA and SEB in raw cow milk. The dot-blot assay exhibited superior sensitivity, whereas AC-ELISA offered higher specificity. These immunoassays provide viable alternatives to PCR, particularly in resource-limited settings, offering cost-effective and rapid detection of *S. aureus* enterotoxins. Further refinement and validation with larger sample sizes are necessary to enhance diagnostic accuracy and minimize cross-reactivity.

## INTRODUCTION

*Staphylococcus* aureus is the third major bacterial pathogen that causes foodborne disease globally [1]. This bacterium has the ability to grow and produce different enterotoxins in various food products under different conditions, including tolerance to a wide range of pH, heating, and denaturing agents, making it a common cause of food poisoning [2]. *S. aureus* is the main bacterial causative agent of both clinical and sub-clinical mastitis in dairy herds [3, 4]. The growth of this bacterium in milk and its products may occur at the onset of preservation processes and production [5].

To date, 24 staphylococcal enterotoxins have been described based on their antigenicity (staphylococcal enterotoxins A (SEA) to staphylococcal enterotoxin-like Y (SElY)) [6]. Among these enterotoxins, SEA and staphylococcal enterotoxin B (SEB) are the most common serotypes involved in staphylococcal food poisoning [7]. Low quantities and concentrations of SEA (200 ng/kg human body weight) may cause food poisoning symptoms such as vomiting, and SEB (100–200 ng; 0.5 ng/mL) in food is sufficient to cause illness. Dairy products are frequently involved in food poisoning, with enterotoxins as low as 0.5 ng/mL [8]. The predominance of SEA and SEB in the immunological detection of the five classical staphylococcal enterotoxins is most frequently associated with staphylococcus food poisoning outbreaks in more than 80% of all countries [9].

A rapid, accurate, and sensitive test method for detecting the presence of low-level staphylococcal enterotoxins in milk and food is urgently needed to assess the safety of food commodities and confirm the diagnosis in cases of food poisoning. A rapid detection tool is needed to detect enterotoxins at low concentrations. Milk is easily exposed to staphylococcus as these organisms are common in the udder and may be induced in cows [5]. In Staphylococcal food poisoning, *S. aureus* produces enterotoxins that contaminate raw milk. To examine outbreaks caused by staphylococcal enterotoxins in milk, the best method is to directly detect the poisoning agent, the enterotoxins [10]. Immunoassay methods can be developed and used to analyze the presence of toxins in milk inoculated with Enterotoxigenic *Staphylococcus aureus* strains [11].

Enzyme-linked immunosorbent assay (ELISA) is an immunological method that involves an enzyme to detect antigens in a sample, as well as to examine humoral immune responses [12]. The ELISA method was developed as a biosensor based on the colorimetric principle as part of enzyme immunoassay. This method is very sensitive and can detect low concentrations of staphylococcal enterotoxins in milk [5, 13]. Based on the principles of an ELISA that relies on antibodies to detect a target antigen using specific antigen-antibody interactions, an indirect AC-ELISA method was developed for detecting SEA and SEB as targeting antigens in raw cow milk samples. This assay uses polyclonal antibodies as the capture antibodies [14] and a goat anti-mouse Alkaline Phosphatase (AP) conjugate as the detection system. The assay allows for easy visualization of results and can be completed without the use of additional radioactive materials. ELISA has become an increasingly important technique for detecting food poisoning agents [15].

Although *S. aureu*s is well known for producing enterotoxins that contribute to foodborne illnesses, the detection of SEA and SEB in raw cow milk remains a challenge. Molecular techniques such as polymerase chain reaction (PCR) can identify the presence of enterotoxin-encoding genes but do not confirm the direct presence of the enterotoxins themselves. This creates a critical gap in food safety monitoring, where an effective, sensitive, and rapid diagnostic tool is necessary for detecting these toxins in dairy products before they pose a risk to public health. Existing immunoassays such as ELISA and dot-blot assays have not been extensively compared to molecular methods for their diagnostic reliability in detecting SEA and SEB in raw milk. In addition, there is limited research evaluating the sensitivity, specificity, and agreement of immunoassays with PCR in resource-limited settings.

This study aims to develop and evaluate two immunoassays – antigen capture ELISA (AC-ELISA) and dot-blot Assay – for the detection of SEA and SEB in cow milk. The diagnostic performance of these immunoassays will be assessed in comparison to the standard PCR method. By analyzing sensitivity, specificity, and overall agreement with PCR, this research seeks to determine the feasibility of using these immunoassays as alternative diagnostic tools for food safety monitoring, particularly in resource-limited settings.

## Materials and Methods

### Ethical approval

All procedures performed on animals in this study complied with the ethical clearance issued by the Animal Ethics Committee of Universitas Gadjah Mada (Approval no. 048/EC-FKH/Int./2023).

### Study period and location

The study was conducted from September 2023 to September 2024 at Clinical Pathology Laboratory, Faculty of Veterinary Medicine, Universitas Gadjah Mada.

### Samples collection

A total of 30 raw milk samples were collected aseptically from a traditional dairy farm in Boyolali, Central Jawa, using 50 mL sterile conicle to avoid contamination. The samples were placed in an ice box at 4°C and were immediately transferred to the Laboratory of Clinical Pathology, Universitas Gadjah Mada. The samples were then aliquots in several 15 mL sterile falcon and stored at a temperature below −40°C to ensure the raw milk remained safe and fresh. The samples were centrifuged at 2,500× *g* for 5 min and kept at a temperature below 5°C. Subsequently, the fatty layers of the surfaces were removed and stored at a temperature below −40°C.

### Bacterial culture

In the raw cow milk sample, the stored raw cow milk with non-fatty layers was thawed at room temperatur*e* (24°C). The samples were inoculated onto Mannitol Salt Agar (MSA) plates by streaking and incubating for 24 h at 37°C. This medium is specific to staphylococcal species, allowing them to ferment mannitol into acid. Isolates of *S. aureus* grew as round colonies with a characteristic medium golden yellow color with a yellow zone. The suspected *S. aureus* colonies were clarified using Gram staining, slide coagulase, and catalase. A single colony suspected of *S. aureus* was transferred on Brain Heart Infusion Broth (BHI-B) containing 1% yeast extract and incubated for 24 h at 37°C for genomic DNA isolation [16].

### DNA extraction

Genomic DNA was extracted from raw milk that was extracted using the Geneaid™ DNA isolation kit (Taipei, Taiwan) according to the manufacturer’s protocol. A total of 1000 μL bacterial culture in BHI-B were transferred to a 1.5-microcentrifuge tube and were pelleted at approximately 14,000× *g* for 1 min. The supernatant was removed and washed by adding 100 μL sterile phosphate-buffered saline (PBS). The suspension was centrifuged at 12,000× *g* for 10 min at 10°C and the supernatant was discarded. The pellets were transferred to 200 μL Gram+ buffer containing 4 μL lysostaphin (1.8 U/μL; Sigma, USA) and vortexed completely. The suspension was incubated at 37°C for 30 min and the tube was inverted every 10 min, followed by the addition of 20 μL of proteinase K (14.8 mg/mL; Sigma). The suspension was incubated at 60°C for 10 min and was inverted the tube every 2–3 min. We added the suspension with 200 μL GB buffer (Genaid) and vortex for 2 s. The suspension was incubated at 70°C for 10 min with shaking every 3 min. Transferred the supernatant to a new 1.5 mL centrifuge tube, and then added 200-μL isopropanol. The centrifuge was used at 14,000× *g* for 2 min, and the aqueous supernatant was transferred to a spin column. A total of 400 μL W1 buffer was added to each sample, and the suspensions were at 14,000× *g* for 30 s and the supernatant was discarded. The spin columns were placed in a clean collection tube. Washed the pellet by adding 600 μL wash buffer and centrifuged at 12,000× *g* for 30 s. The supernatant was discarded in the collection tube with the flow through. The suspensions were centrifuged at 14,000× *g* for 3 min, and the spin column was placed in a clean 1.5 mL microcentrifuge tube. The DNA was eluted using 100 μL of preheated elution buffer at 70°C. After adding the buffer, the column was incubated for 3 minutes at 24°C. The suspensions were centrifuged at 14,000× *g* for 3 min, and the spin column. The extracted DNA was detected by electrophoresis on 1% agarose gel and was stored at −20°C.

### PCR amplification

The detection of *S. aureus* enterotoxins encoding *sea* and *seb* genes was assessed using PCR. Genotyping was performed based on the presence of 23S ribosomal RNA (rRNA) genes, *nuc*, and *coa* gene specific to *S. aureus*. Amplification reactions were performed in a final volume of 25 μL containing 12.5 μL 2× My Taq HS red mix (Bioline, United Kingdom), 10.5 μL (ddH_2_O, 2.0 mM primer pair), and 2 μL DNA template. The primers and programs used for *S. aureus* genes (*23S rRNA*, *nuc*, and *coa* genes) and enterotoxins genes (*sea* and *seb* genes) are listed in Table 1 [1, 17].

**Table 1:** Nucleotide sequences of the oligonucleotide primers used in gene amplification.

Target gene	Oligonucleotide sequence (5’➔3’)	Size (bp)	Program	References
*23S rRNA*	ACGGAGTTACAAAGGACGAC AGCTCAGCCTTAACGAGTAC	1250	1*	[1]
*nuc*	GCGATTGATGGTGATACGGTT ACGCAAGCCTTGACGAACTAAAGC	279	2*	[1]
*coa*	ATAGAGATGCTGGTACAGG GCTTCCGATTGTTCGATGC		3*	[1]
*sea*	CCTTTGGAAACGGTTAAAACG TCTGAACCTTCCCATCAAAAAC	127	4*	[17]
*seb*	TCGCATCAAACTGACAAACG GCAGGTACTCTATAAGTGCCTGC	477	5*	[17]

1* 37 cycles of 94°C for 5 min, 94°C for 40 s, 64°C for 60 s, 72°C for 75 s, 72°C for 5 min; 2* 37 cycles of 94°C for 5 min, 94°C for 1 min, 55°C for 30 s, 72°C for 30 s, 72°C for 5 min; 3* 30 cycles of 94°C for 5 min, 94°C for 1 min, 58°C for 1 min, 72°C for 1 min, and 72°C for 5 min; 4* 35 cycles: 95°C for 15 min, 95°C for 30 s, 57°C for 90 s, 72°C for 90 s, 72°C for 10 min; 5* 35 cycles, 95°C for 15 min, 95°C for 30 s, 57°C for 90 s, 72°C for 90 s, 72°C for 10 min, rRNA=Ribosomal RNA

The amplification reaction was performed using a TC 9639 Thermal Cycle instrument (Benchmark Scientific, USA). The PCR products were resolved by electrophoresis in 1.5% agarose gel (Invitrogen, USA) in 1× tris-borate-ethylenediaminetetraacetic acid buffer, stained by 0.5 μg/mL RedSafe Nucleic Acid Staining Solution (iNtRon biotechnology, Korea), and visualized on an ultraviolet transilluminator (Optima Inc., Tokyo, Japan).

### Optimization of the ELISA assays

A checkerboard titration was performed to determine the optimal dilution factor for the antigen coating. Several concentrations and dilutions of recombinant protein were used as antigen capture and pasteurized milk was used as a negative control. The amount of lysate coated was tested at different concentrations ranging from 2.5 μg/mL, 5 μg/mL, 7.5 μg/mL, 10 μg/mL, and 15 μg/mL based on the dot-blot assay’s result, the limit concentration that showed a dark pattern.

### Determination of the cutoff value for ELISA

To determine the cutoff point for the assay, 36 samples from previous study were used, negative samples were analyzed in the assay, and the upper limit of mean + (2× standard deviation) value was determined as the cutoff point.

### AC-ELISA of raw cow milk samples

AC-ELISA was performed using a 96-well maxisorp immunoplate (Nunc-immunoplate, Maxisorp, Roskilde, Denmark) through indirect methods. The plate was coated with 100 μL/well filtered raw cow milk samples and incubated overnight at 37°C. The next day, the plate was washed 3× with wash buffer (PBS containing 0.05% tween-20, pH 7.2). Afterward, each well was blocked by 200 μL of blocking solution (5% dry milk in 0.01 M PBS with 0.05% Tween-20) and incubated for a minimum of an hour at 37°C and was flicked off 3× with wash buffer. The wells were added with paired rows of 100 μL polyclonal antibody of SEA or SEB diluted at 1:3000 with incubation solution with pH 7.2 and incubated at 37°C for an hour. The plate was subjected to 3× washes with washing buffer. A volume of 100 μL/well of commercial AP (Sigma) conjugated goat anti-mouse immunoglobulin G (IgG) (1:5000 dilution in incubating solution). The reaction was conducted at 37°C for an hour. Finally, the plate was washed 3× and was added with 100 μL/well substrate 4-nitrophenyl phosphate (Merck, USA) for 30 min. Absorbance was measured at 405 nm using a multimode microplate reader (BioTek, Germany). Each test sample was tested in a duplet and the average absorbance values were statistically analyzed.

### Optimization of the dot-blot assay

To determine the minimum concentration of antigen for SEA and SEB, serial dilutions of antigen SEA and SEB concentrations ranging from 1 μg/mL, 2.5 μg/mL, 5 μg/mL, 7.5 μg/mL, 10 μg/mL, 25 μg/mL, 50 μg/mL, 75 μg/mL, and 100 μg/mL were tested with PBS as a negative control. The dots with dark patterns at the lowest concentration were used as positive controls. Dots with intensity similar to or greater than the positive control dot pattern are positive; otherwise, a lower dot pattern is negative.

### Dot-blot assay to detect SEA and SEB in raw milk samples

The dot-blot test was performed on a nitrocellulose membrane (NCM) with 0.2 μm pore size (Bio-Rad, USA). A volume of 5 μL of raw milk sample was dropped onto a paper sheet until well dried. Subsequently, the samples were added with blocking buffer 1% bovine serum albumin in 0.5% tris-buffered saline with Tween 20 (TBST) and were incubated at 37°C an hour in an incubator shaker. The samples were flicked off 3× intervals 5 min with wash buffer (0.05% TBST). The polyclonal antibody produced in mice [18] diluted to 1:3000 in incubation solution (pH 7.2) was added to the plate and incubated at 37°C for 1 h in a shaking incubator. The plate was subjected to 3× washes followed by drops of 10 mL of AP (Sigma) conjugated goat anti-mouse IgG diluted 1:5000 in incubation solution. Subsequently, the samples were flicked off 2× intervals 5 min with wash buffer (0.05% TBST) and 1× with buffer tris-buffered saline. Finally, the plate was stained using nitro-blue tetrazolium and 5-bromo-4-chloro-3’-indolyphosphate (Sigma) and terminated with ddH_2_O.

### Statistical analysis

The diagnostic performance of AC-ELISA and dot-blot assays in detecting SEA and SEB in raw cow milk was evaluated through statistical analysis. Sensitivity and specificity were calculated to determine the ability of each assay to correctly identify positive and negative cases, with PCR serving as the reference standard. The positive predictive value (PPV) and negative predictive value (NPV) were also computed to assess the likelihood of true positive and true negative results, respectively.

The overall accuracy of each assay was measured as the proportion of correctly classified samples among the total tested samples. Cohen’s Kappa coefficient (κ) was employed to assess the agreement between the AC-ELISA, dot-blot assays, and PCR results. The κ values were interpreted using standard criteria, ranging from poor agreement (<0.20) to almost perfect agreement (0.81–1.00). In addition, likelihood ratios for positive and negative results were determined to estimate the diagnostic strength of each assay.

Statistical analyses were performed using MedCalc statistical software (MedCalc Software Ltd., Belgium) for diagnostic test evaluation, as well as the Evidence-Based Medicine Toolbox (EBM-Tools, Toronto, Canada). Sensitivity, specificity, PPV, and NPV were reported with 95% confidence intervals (CIs) to ensure statistical reliability. Comparative analyses were conducted to assess the performance of the AC-ELISA and dot-blot assays against the established PCR method in detecting SEA and SEB.

## RESULTS

### Bacterial culture of raw cow milk

All 30 raw milk samples were cultured on MSA to screen for *S*. *aureus*. Suspected *S. aureus* colonies appeared as round, golden-yellow colonies with yellow zones on a selective medium. Isolated colonies were examined for morphological characteristics using Gram staining. Biochemical tests, including catalase and coagulase tests, were performed to confirm *S. aureus* identification. Phenotypic analysis detected *S. aureus* in 27–30 samples (90%), whereas 10% of the samples were positive for *Streptococcus* spp.

### PCR assay of the *S. aureus* gene

Genotyping was performed using PCR to detect specific 1250-bp DNA fragments of the *23S rRNA*, *nuc*, and *coa* genes associated with *S. aureus*. Based on molecular amplification, 27 of the 30 samples (90%) were identified as *S. aureus*. These 27 *S. aureus-*positive raw milk samples were further analyzed using both the ELISA and dot-blot assays.

### Optimized AC-ELISA of the captured antigens (recombinant SEA [rSEA] and recombinant SEB [rSEB])

**Figure 1:**
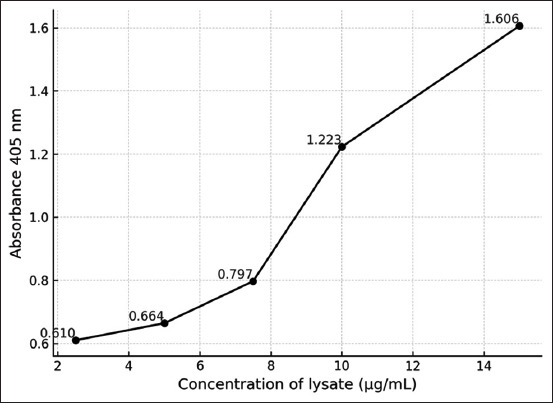
Minimum dilutions (pasteurized milk spike rSEB) samples that tested positive were still detectable at ranging concentrations of 2.5 μg/mL, 5 μg/mL, 7.5 μg/mL, 10 μg/mL, and 15 μg/mL showed OD ≥ 0.610, OD ≥ 0.664, OD ≥ 0.797, OD ≥ 1.223, and OD ≥ 1.606. OD=Optical density, rSEB=Recombinant staphylococcal enterotoxins B.

Based on the optimization, the inhibition capture antigen-rSEA and rSEB binding with lysate coated at 2.5 μg/mL, 5 μg/mL, 7.5 μg/mL, 10 μg/mL, and 15 μg/mL showed optical density (OD) ≥ 0.833, OD ≥ 0.914, OD ≥ 1.141, OD ≥ 1.393, and OD ≥ 1.612, and OD ≥ 0.610, OD ≥ 0.644, OD ≥ 0.797, OD ≥ 1.223, and OD ≥ 1.606, respectively. The data confirmed that the minimum concentration of rSEA (10 μg/mL) and rSEB (7.5 μg/mL) lysate was probed in NCM at OD ≥ 1.141 and OD ≥ 0.797, respectively. The titration results are presented in Figure 1.

### Cutoff values for the ELISA assays

The data show that the cutoff points of SEA and SEB were 0.810 and 0.527, respectively. All the sample’s value ≥0.810 was considered positive for SEA, and all the sample’s values ≥0.527 were examined to be positive for SEB.

### ELISA of raw cow milk samples

The ELISA analysis identified 27 raw cow milk samples. Of these, seven samples were determined to be positive for SEA and SEB, exhibiting OD values equal to or exceeding the respective cutoff points. In contrast, twenty samples displayed OD values below the cutoff threshold and were considered negative. The ELISA results were subsequently compared with the established PCR assay for detecting the *sea* and *seb* genes.

### Optimization of the dot-blot assay

**Figure 2:**
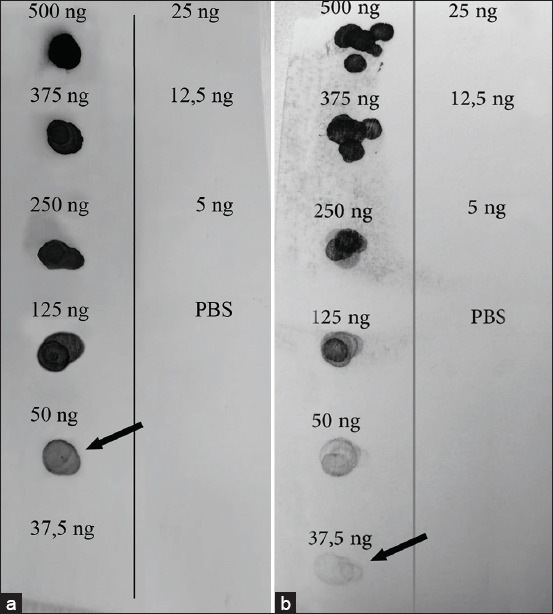
(a) Dot-blot assay for rSEA dilution and PBS as negative control. (b) rSEB dilution and PBS as negative control. The number indicating the quantity of the sample (dropped 5 μL) was recorded. The arrow indicates the minimum concentration to detect SEA was 10 μg/mL (50 ng) and SEB was 7.5 μg/mL (37.5 ng). rSEA=Recombinant staphylococcal enterotoxins A, rSEB=Recombinant staphylococcal enterotoxins B, PBS=Phosphate-buffered saline.

Based on the optimization, the concentration of 10 μg/mL of lysate exhibited a dark pattern at the lowest concentration of SEA and 7.5 μg/mL of SEB. The minimum concentrations required to detect SEA and SEB were 50 ng and 37.5 ng, respectively. The reactivity of the samples is shown in Figure 2. The illumination of the dot pattern showed the level of lysate concentration and quantities.

### Dot-blot assay of raw milk samples

The dot-blot assay was used to analyze SEA and SEB from 27 raw milk samples. Of a total of 27 samples, nine samples were estimated positive for SEA, and 10 were positive for SEB by showing a dark pattern on the membrane with intensity similar to or greater than that of the positive control dot. Out of the 27 samples, 18 SEA were predicted to be negative and SEB was predicted to be negative in 17 samples with the absence of a dark spot in the NCM. The results were then compared with those of the established *sea* gene using PCR methods. Comparison of the dot-blot assays with the established PCR test for the *sea* and *seb* gene.

### Detection of the sea and seb genes

The 27 isolates were identified for staphylococcal enterotoxins encoding *sea* and *seb* genes. At this stage, a 127-bp fragment indicates the *sea* gene, and a 477-bp fragment indicates the *seb* gene. Among the 26 *S. aureus* isolates, 7 isolates (23.3%) harbored the *sea* gene and 8 isolates (30.8%) carried the *seb* gene. Furthermore, 2 samples (7.7%) were encoding a combination of two enterotoxins *se* (*a* and *b*).

The performance of the AC-ELISA indirect methods was compared with that of the established PCR assays. The assay identified five of the seven positive samples, with two samples being misclassified (false negative). Out of the 20 negative samples two samples were misclassified (false positive) of SEA. SEB was correctly identified; six of the eight positive samples with two samples were misidentified (false negative), and 19 samples were negative with two samples being misclassified (false positive). The results of the dot-blot assay were compared with those of the PCR assay. The data of SEA were identified in six of the seven positive samples, with one sample being misclassified (false negative). In contrast, 20 negative samples with three samples were misclassified (false positive). The reactivity for SEB was shown in seven of the eight positive samples, with only one sample which was misidentified (false negative). Three of the 19 negative samples were misclassified (false positive). The comparison of the AC-ELISA and dot-blot assays with the established PCR test of *sea* gene and *seb* gene is shown in Table 2.

**Table 2:** Comparison of AC-ELISA and PCR assays for raw milk samples.

Parameter	AC-ELISA		Dot-blot assays	
	
	SEA	SEB	SEA	SEB
Number of tested samples	27	27	27	27
Number of truly positive	5	6	6	7
Number of truly negative	18	17	17	16
Number of false positive	2	2	3	3
Number of false negative	2	2	1	1

AC-ELISA=Antigen capture enzyme-linked immunosorbent assay, PCR=Polymerase chain reaction, SEA=Staphylococcal enterotoxins A, SEB=Staphylococcal enterotoxins B

### Statistical analysis

**Figure 3:**
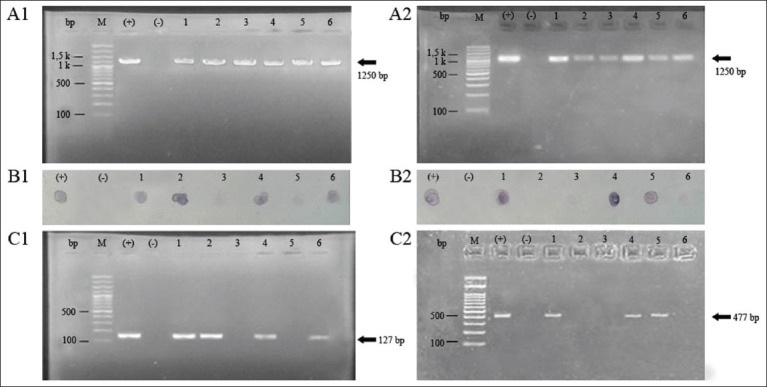
Comparison of SEA and SEB results using dot-blot assays and PCR for *23S rRNA* genes specific to *Staphylococcus aureus* and *sea*, and *seb* gene. (A1 and A2) PCR results for *23S rRNA* genes (1250 bp). (B1 and B2) The NCM was probed with the samples for SEA and SEB. (C1 and C2) Comparison of the assays to the established PCR methods for *sea* gene (127 bp) and *seb* gene (477 bp). SEA=Staphylococcal enterotoxins A, SEB=Staphylococcal enterotoxins B, PCR=Polymerase chain reaction, rRNA=Ribosomal RNA.

According to the optimization of the cutoff value, the performance of the ELISA of SEA and SEB was calculated. The ELISA results showed sensitivity and specificity for SEA at 0.714 (95% CI: 35.90–91.80) and 0.900 (95% CI: 69.90–97.20). The sensitivity and specificity of SEB were calculated as 0.750 (95% CI: 40.90–92.90) and 0.895 (95% CI: 68.60–97.10). The sensitivity and specificity of the dot-blot assay for SEA were 0.857 (95% CI: 48.7–97.4) and 0.850 (95% CI: 64.00–94.8). The dot-blot of SEB shows sensitivity and specificity at 0.857 (95% CI: 52.9–97.8) and 0.842 (95% CI: 62.4–94.5). The sensitivity and specificity of the assays are presented in Table 3. The performance of the dot-blot assay compared with the result of the established PCR assay and the analysis of the result is presented in Figure 3.

**Table 3:** Diagnostic parameters of the ELISA and dot-blot assays.

Diagnostic parameters	Estimated value	

	SEA	SEB
AC-ELISA		
Sensitivity (95% CI)	71.40 (35.90–91.80)	75.00 (40.90–92.90)
Specificity (95% CI)	90.00 (69.90–97.20)	89.50 (68.60–97.10)
Positive likelihood ratio (95% CI)	7.14 (1.76–28.84)	7.14 (1.81–28.06)
Negative likelihood ratio (95% CI)	0.32 (0.09–1.03)	0.28 (0.08–0.94)
Positive predictive value (*) (%)	71.40	75.00
Negative predictive value (*) (%)	90.00	89.50
Accuracy (*) (%)	72.36	75.72
κ (95% CI)	0.61 (0.27–0.96)	0.64 (0.33–0.96)
SE of κ	0.162	0.162
Dot-blot assay		
Sensitivity (95% CI)	85.70 (48.7–97.4)	87.50 (52.9–97.8)
Specificity (95% CI)	85.00 (64.00–94.8)	84.20 (62.4–94.5)
Positive likelihood ratio (95% CI)	5.71 (1.93–16.90)	5.53 (1.89–16.17)
Negative likelihood ratio (95% CI)	0.17 (0.03–1.04)	0.14 (0.02–0.93)
Positive predictive value (*) (%)	66.71	70.00
Negative predictive value (*) (%)	94.4	94.10
Accuracy (*) (%)	85.68	87.34
κ (95% CI)	0.65 (0.33–0.96)	0.67 (0.37–0.96)
SE of κ	0.159	0.149

AC-ELISA=Antigen capture enzyme-linked immunosorbent assay, SEA=Staphylococcal enterotoxins A, SEB=Staphylococcal enterotoxins B, CI=Confidence interval, SE=Standard error

The predictive value considered as a positive-test probability revealed a PPV of AC-ELISA for SEA and SEB of 71.4% and 75.0%. The PPVs of the dot-blot assay of SEA and SEB were 67.7% and 70.0%. This shows the proportion of true positives among all samples with a positive test. On the other hand, the proportion of true negatives among all samples with a negative test was considered the NPV. In the ELISA of SEA and SEB, the NPVs were calculated as 90.0% and 89.5%. The dot-blot assay results were shown for 94.4% and 94.1%.

The accuracy data of the AC-ELISA of SEA and SEB were 72.36% and 75.72%. The dot-blot assay showed a SEA and SEB of 85.68% and 87.34%, respectively. The accuracy estimate shows the proportion between true-positive and true-negative results in relation to all possible results. Both assays were compared with the established PCR methods with a Kappa index. The values were 0.61 and 0.64 for ELISA SEA and SEB, respectively. The dot-blot assays of SEA and SEB showed values of 0.65 and 0.67. These results exhibited that the Kappa value falls under the interpretation of substantial agreement (0.61–0.80), where the kappa index 1 is a perfect agreement.

## Discussion

This study was designed to determine the presence of antigens against SEA and SEB in raw cow milk using the AC-ELISA and dot-blot assays, which were then compared with the presence on PCR methods. The performance optimization of the dot-blot assays was recognized for SEA at the lowest concentration of 10 μg/mL and SEB of 7.5 μg/mL, showing a dark pattern on a membrane with the lysate concentrations of 50 ng (SEA) and 37.5 ng (SEB). In this case, the volume of lysate dropped into a single well was 5 μL. In general, the toxin concentrations in food vary from 0.05 ng/g to 25 ng/g, and in dairy products, enterotoxins are frequently present at levels as low as 0.5 ng/g [10]. As low as quantities (100–200 ng) and concentrations (0.5–1 ng/g), SEs can lead to food intoxication and cause diseases with classic food poisoning symptoms (nausea, vomiting, and diarrhea) [19].

The results show that both assays have convincing sensitivity and high specificity for SEA and SEB detection compared with the established PCR method. The sensitivity for the ELISA was determined at 71.40% (35.9–91.8) of SEA and 75.00% (40.9–92.9) of SEB, whereas the dot-blot assay had a slightly higher estimation at around 85.70% (48.7–97.4) of SEA and 87.50% (52.9–97.8) of SEB. Both assays showed a specificity of around 84%–90%, with the specificity of the ELISA assay being slightly higher than that of the dot-blot assay. The AC-ELISA assay results demonstrated the capability of polyclonal antibodies to detect enterotoxins in raw cow milk.

In general, the results of our study demonstrated that the AC-ELISA and dot-blot assays did not significantly differ in diagnostic sensitivity compared with the established PCR method for detecting SEA and SEB based on the recommended cut-off value. Furthermore, both assays displayed higher specificity (80%–90%), implying that the ELISA and dot-blot assays are applicable for precisely recognizing SEA and SEB. Moreover, both assays exhibited Kappa values that fell under the interpretation of substantial agreement (0.61–0.80), suggesting that the AC-ELISA and dot-blot assays are promising methods for detecting the presence of enterotoxins of SEA and SEB in raw cow milk.

Although molecular techniques such as PCR have been widely used to detect the existence of staphylococcal enterotoxins, this method requires sophisticated equipment and high-cost laboratory settings and cannot directly detect enterotoxins and assess their levels in food. The AC-ELISA and dot-blot assay show that a sensitivity score >70% would be considered developing, a specificity score >80% is good, and an accuracy score >70% is considered acceptable for diagnostic measures.

AC-ELISA has the advantage of being more specific regarding the number of ODs on the samples. On the other hand, the dot-blot assay is easier and simpler to perform and requires no specific equipment. Thus, the assay can be used as a preliminary screening for the existence of SEA and SEB, particularly in low-resource settings with limited laboratory facilities, and ELISA can be used where laboratory facilities are available.

## CONCLUSION

This study evaluated the diagnostic performance of AC-ELISA and dot-blot assays for detecting SEA and SEB in raw cow milk, comparing them with the PCR method as the reference standard. The results demonstrated that both immunoassays exhibited considerable diagnostic potential, with the dot-blot assay demonstrating higher sensitivity (85.7% for SEA and 87.5% for SEB) compared to AC-ELISA (71.4% for SEA and 75% for SEB). In contrast, AC-ELISA exhibited slightly superior specificity (90.0% for SEA and 89.5% for SEB) compared to the dot-blot assay (85.0% for SEA and 84.2% for SEB). Statistical analysis confirmed a substantial agreement between both immunoassays and PCR (κ values of 0.61–0.67), reinforcing their reliability as diagnostic tools.

This study offers a valuable comparative evaluation of diagnostic assays for SEA and SEB detection, highlighting their practical advantages over PCR. The immunoassays provide rapid, cost-effective, and accessible alternatives, particularly for resource-limited settings, where molecular methods may not be feasible. The high sensitivity and specificity observed in both assays underscore their potential for routine food safety monitoring and early detection of enterotoxin contamination in dairy products. However, the study has certain limitations, including a relatively small sample size, which may affect the generalizability of the findings. In addition, while specificity was high, the potential for cross-reactivity with other staphylococcal enterotoxins remains a concern. Another limitation is the qualitative nature of the dot-blot assay, which relies on visual interpretation and may introduce variability in result analysis.

To enhance the robustness of these findings, future studies should incorporate larger sample sizes across diverse dairy production regions to improve statistical reliability. Expanding the detection spectrum to include other staphylococcal enterotoxins, such as staphylococcal enterotoxin C, staphylococcal enterotoxin D, and staphylococcal enterotoxin E, could further strengthen diagnostic coverage. In addition, the development of quantitative dot-blot assays using image-based or automated analysis could improve result standardization. Integrating these immunoassays into multiplex diagnostic platforms would enable simultaneous detection of multiple foodborne pathogens, increasing their applicability in food safety monitoring. Field validation in dairy supply chains is also necessary to assess their feasibility for routine screening in real-world settings.

Overall, this study highlights the potential of immunoassay-based detection methods as viable alternatives to molecular techniques for monitoring *S. aureus* enterotoxin contamination in dairy products. With further optimization and validation, these assays could be crucial in ensuring food safety, mitigating the risks associated with Staphylococcal food poisoning, and strengthening public health protection measures.

## AUTHORS’ CONTRIBUTIONS

HNP: Performed the experiment, data analysis, and wrote the manuscript. SIOS: Conceptualization, funding acquisition, supervised the study, and drafted the manuscript. FA and MW: Data analysis and reviewed the manuscript. RW: Data analysis and drafted the manuscript. All authors have read and approved the final manuscript.
